# Rivaroxaban for the treatment of symptomatic deep-vein thrombosis and pulmonary embolism in Chinese patients: a subgroup analysis of the EINSTEIN DVT and PE studies

**DOI:** 10.1186/1477-9560-11-25

**Published:** 2013-12-16

**Authors:** Yuqi Wang, Chen Wang, Zhong Chen, Jiwei Zhang, Zhihong Liu, Bi Jin, Kejing Ying, Changwei Liu, Yuxia Shao, Zhicheng Jing, Isabelle Ling Meng, Martin H Prins, Ákos F Pap, Katharina Müller, Anthonie WA Lensing

**Affiliations:** 1 Department of Vascular Surgery, Zhongshan Hospital, Fudan University, Shanghai, China; 2 Beijing Institute of Respiratory Medicine Beijing Chaoyang Hospital, Beijing, China; 3 Department of Vascular Surgery Beijing Anzhen Hospital, Beijing, China; 4 Renji Hospital, School of Medicine, Shanghai Jiao Tong University, Shanghai, China; 5 Department of Cardiology, Cardiovascular Institute & Fu Wai Hospital, Beijing, China; 6 Department of Surgery Wuhan Union Hospital, Affiliated to Tongji Medical College, Huazhong University of Science and Technology, Wuhan, China; 7 Department of Respiratory Medicine, Sir Run Run Shaw Hospital, Zhejiang University School of Medicine, Affiliated with School of Medicine, Zhejiang University, Hangzhou, China; 8 Department of General Surgery, Peking Union Medical College Hospital, Beijing, China; 9 Department of Respiratory Medicine, The 2nd Affiliated Hospital of Harbin Medical University, Harbin, China; 10 Department of Pulmonary Circulation, Tongji University Affiliated Shanghai Pulmonary Hospital, Shanghai, China; 11 Thrombosis and Vascular Medicine Center, State Key Lab of Cardiovascular Disease, Fu Wai Hospital, Peking Union Medical College and Chinese Academy of Medical Science, Beijing, China; 12 Bayer HealthCare, Berlin, Germany; 13 Maastricht University, Maastricht, The Netherlands; 14 Bayer HealthCare, Aprather Weg 18a, Wuppertal D-42096, Germany

**Keywords:** Rivaroxaban, Deep vein thrombosis, Pulmonary embolism, Venous thromboembolism, Vitamin K antagonist, Randomized trial

## Abstract

**Background:**

The worldwide EINSTEIN DVT and EINSTEIN PE studies randomized 8282 patients with acute symptomatic deep-vein thrombosis (DVT) and/or pulmonary embolism (PE) and, for the first time in trials in this setting, included patients in China. This analysis evaluates the results of these studies in this subgroup of patients.

**Methods:**

A total of 439 Chinese patients who had acute symptomatic DVT (n=211), or PE with or without DVT (n=228), were randomized to receive rivaroxaban (15 mg twice daily for 21 days, followed by 20 mg once daily) or standard therapy of enoxaparin overlapping with and followed by an adjusted-dose vitamin K antagonist, for 3, 6, or 12 months. The primary efficacy outcome was symptomatic recurrent venous thromboembolism. The principal safety outcome was major or non-major clinically relevant bleeding.

**Results:**

The primary efficacy outcome occurred in seven (3.2%) of the 220 patients in the rivaroxaban group and in seven (3.2%) of the 219 patients in the standard-therapy group (hazard ratio, 1.04; 95% confidence interval 0.36–3.0; p=0.94). The principal safety outcome occurred in 13 (5.9%) patients in the rivaroxaban group and in 20 (9.2%) patients in the standard-therapy group (hazard ratio, 0.63; 95% confidence interval 0.31–1.26; p=0.19). Major bleeding was observed in no patients in the rivaroxaban group and in five (2.3%) patients in the standard-therapy group. In fragile patients (defined as age >75 years, creatinine clearance <50 mL/min, and/or body weight ≤50 kg), the principal safety outcome occurred in four (8.9%) of the 45 patients who received rivaroxaban compared with seven (15.2%) of the 46 patients who received standard therapy.

**Conclusions:**

In Chinese patients with acute symptomatic DVT and/or PE, rivaroxaban was as efficacious as enoxaparin followed by vitamin K antagonist therapy, with a similar safety profile. The relative efficacy and safety of rivaroxaban compared with enoxaparin/vitamin K antagonist were consistent with that found in the rest of the world.

**Trial registration number:**

EINSTEIN PE, ClinicalTrials.gov NCT00439777; EINSTEIN DVT, ClinicalTrials.gov NCT00440193

## Background

Acute venous thromboembolism (VTE; i.e. deep-vein thrombosis [DVT] or pulmonary embolism [PE]) is a common disorder with an annual incidence in the Western world of approximately 1–2 cases per 1000 persons in the general population [1,2]. Short-term treatment of VTE is effective and reduces the risk of recurrent disease, which is the major complication, from an estimated 25% to approximately 3% during the first 6–12 months of therapy [3]. However, the risk of recurrent VTE remains after treatment ends and can reach 5–10% during the first year [4,5].

Although the incidence of VTE in China is not known, the disease is increasingly being recognized by the Chinese healthcare system; this trend may arise as a result of increasing physician attention and the availability of minimally invasive and non-invasive diagnostic tools. The results of VTE treatment among Chinese nationals are not well documented, although there seems to be a general belief that the risk of bleeding is high among patients receiving standard treatment, and that vitamin K antagonist (VKA) therapy should be dosed carefully with a tendency towards lower international normalized ratio (INR) values. Similar concerns about bleeding would also apply to the direct oral anticoagulants.

Rivaroxaban is an orally active, direct Factor Xa inhibitor with a rapid onset of action and predictable pharmacokinetics and pharmacodynamics [6], which does not require routine coagulation monitoring, has no food interactions, and has limited drug interactions. These properties have also been confirmed through a rivaroxaban dose escalation study in healthy, elderly Chinese subjects [7]. The EINSTEIN DVT and EINSTEIN PE studies evaluated rivaroxaban for the treatment of VTE. In this large, international, phase III clinical program in more than 8000 patients with acute symptomatic DVT and/or PE, monotherapy with rivaroxaban was shown to be as effective as dual-drug therapy with enoxaparin overlapping with and followed by VKA therapy, with reduced occurrence of major bleeding [8]– [11]. In these studies, for the first time, Chinese hospitals participated using the same rivaroxaban regimen.

Here, we report the results of the 439 patients who participated in the EINSTEIN DVT and EINSTEIN PE studies in China.

## Methods

### Study design

EINSTEIN DVT and EINSTEIN PE were randomized, open-label studies that compared the efficacy and safety of rivaroxaban with standard therapy, consisting of enoxaparin and adjusted-dose VKA, in patients with acute, symptomatic DVT and/or PE [8]– [11]. Patients were eligible if they were of legal age and had objectively confirmed acute, symptomatic DVT and/or PE. Briefly, patients were ineligible to participate if they had received a therapeutic dose of parenteral anticoagulant for more than 48 hours or if they had received more than a single dose of a VKA before randomization; if thrombectomy had been performed, a vena cava filter placed, or a fibrinolytic agent administered for treatment of the current episode; or if they had any contraindication listed in the local labeling of enoxaparin, warfarin, or acenocoumarol. Other criteria for ineligibility were, another indication for a VKA; creatinine clearance (CrCl) <30 mL/min; clinically significant liver disease; active bleeding or a high risk of bleeding contraindicating anticoagulant treatment; childbearing potential without proper contraceptive measures; pregnancy or breast-feeding; or a life expectancy of less than 3 months. Because the metabolism of rivaroxaban is mediated by cytochrome P450 3A4 (CYP3A4), CYP3A4 inhibitors can decrease the metabolism of rivaroxaban, causing an increase in the area under the plasma concentration–time curve (AUC) and the maximum plasma concentration (C_max_); conversely, CYP3A4 inducers can increase the metabolism of rivaroxaban, thus decreasing the AUC and C_max_. Therefore, patients requiring concomitant therapy of a strong inhibitor or inducer of CYP3A4 were also ineligible.

### Randomization and treatment regimens

Randomization was performed with the use of a computerized voice-response system and was stratified according to country and the intended treatment duration (3, 6, or 12 months). The intended duration of treatment was determined by the treating physician before randomization. Patients who were assigned to the rivaroxaban group were given 15 mg twice daily for the first 21 days, followed by 20 mg once daily. Patients who were assigned to the standard-therapy group received enoxaparin at a dose of 1.0 mg/kg of body weight twice daily, and either warfarin or acenocoumarol started within 48 hours after randomization. Enoxaparin was discontinued when the INR was ≥2.0 for 2 consecutive days and the patient had received at least 5 days of enoxaparin treatment. The dose of the VKA was adjusted to maintain an INR of 2.0–3.0. The INR was determined at least once a month. The use of non-steroidal anti-inflammatory drugs and antiplatelet agents was discouraged. Aspirin administered at a dose of no more than 100 mg per day, clopidogrel at a dose of 75 mg per day, or both, were permitted.

### Surveillance and follow-up

Patients were followed for the intended treatment period and were assessed at fixed intervals, which were identical for both treatment arms, using a checklist to elicit information on symptoms and signs of recurrent VTE, bleeding, and adverse events. Patients were instructed to report to the study center immediately if any of these symptoms or signs occurred. In the case of suspected VTE, the protocol required objective testing.

### Outcome assessment

The primary efficacy outcome was symptomatic recurrent VTE, which was defined as a composite of fatal and non-fatal PE or DVT on the basis of criteria that have been described previously. Death was classified as being due to PE, bleeding, or other established diagnoses. PE was considered the cause of death if there was objective documentation of the condition, or if death could not be attributed to a documented cause and PE could not be confidently ruled out. The principal safety outcome was clinically relevant bleeding, which was defined as a composite of major and clinically relevant non-major bleeding, as described previously [8,9]. Bleeding was defined as major if it was clinically overt and: a) associated with a decrease in hemoglobin level of ≥2.0 g/dL; b) led to the transfusion of ≥2 units of red cells; c) was intracranial or retroperitoneal, or occurred in another critical site; or d) contributed to death. Clinically relevant non-major bleeding was defined as overt bleeding that did not meet the criteria for major bleeding, but was associated with medical intervention, unscheduled contact with a physician, interruption or discontinuation of a study drug, or discomfort or impairment of activities of daily life. Predefined secondary outcomes included major bleeding, death from any cause, vascular events (acute coronary syndrome, ischemic stroke, transient ischemic attack, or systemic embolism), and net clinical benefit (which was defined as a composite of the primary efficacy outcome and major bleeding, as assessed in the intention-to-treat population). All suspected outcome events were classified by a central adjudication committee whose members were unaware of the treatment assignments.

### Statistical analysis

The primary efficacy analysis was performed on an intention-to-treat basis with the use of a Cox proportional-hazards model stratified according to the intended duration of treatment and index event (DVT/PE), with adjustment for the presence or absence of cancer at baseline. The population for the safety analysis was defined as all patients who received at least one dose of a study drug. Bleeding events were included in the analysis if they occurred during treatment or within 2 days after the last dose of a study drug. Kaplan–Meier curves were generated to display the distribution of bleeding over time. In addition, analyses of the principal safety outcome in prespecified subgroups were performed. The mean time during which the INR was within the therapeutic range was calculated after the discontinuation of enoxaparin, with correction for interruptions in the administration of VKAs.

## Results

### Patients

From May 2008 through to March 2011, 439 patients were enrolled at 21 sites in China (details of investigators and sites are listed in Additional file 1) 211 patients had DVT only and 228 had PE with (n=67) or without DVT (n=161). A total of 220 patients were assigned to receive rivaroxaban, and 219 were assigned to receive enoxaparin and a VKA (standard therapy). The characteristics of patients were similar at baseline in the rivaroxaban and standard-therapy groups (Table 1). One patient in each treatment arm did not receive the assigned study treatment.

**Table 1:** Demographic and clinical characteristics: Chinese patients in the EINSTEIN DVT and EINSTEIN PE studies*

**Characteristic**	**Rivaroxaban (n=220)**	**Standard therapy (n=219)**
*Mean age, years*	58.6 ± 15.8	59.0 ± 15.0
*Male sex, n (%)*	127 (57.7)	112 (51.1)
*Weight, n (%)*		
≤50 kg	11 (5.0)	15 (6.8)
>50–80 kg	182 (82.7)	188 (85.8)
>80 kg	25 (11.4)	16 (7.3)
Missing data	2 (0.9)	0
*Creatinine clearance, n (%)*		
<30 mL/min	1 (0.5)	0
30–<50 mL/min	19 (8.6)	28 (12.8)
50–<80 mL/min	75 (34.1)	72 (32.9)
≥80 mL/min	117 (53.2)	116 (53.0)
Missing data	8 (3.6)	3 (1.4)
*Diagnostic method, PE cohort, n (%)*		
Spiral computed tomography	99 (86.8)	102 (87.9)
Ventilation–perfusion lung scanning	11 (9.6)	10 (8.8)
Pulmonary angiography	3 (2.6)	1 (0.9)
Not confirmed/not evaluable	1 (0.9)	3 (2.6)
*Anatomical extent of PE, n (%)*		
Limited: ≤25% of vasculature of a single lobe	13 (11.4)	10 (8.6)
Intermediate	67 (58.8)	73 (62.9)
Extensive: multiple lobes and >25% of entire pulmonary vasculature	30 (26.3)	29 (25.0)
Not assessable	4 (3.5)	4 (3.4)
*Diagnostic method, DVT cohort, n (%)*		
Ultrasonography	130 (90.9)	120 (89.6)
Venography	1 (0.7)	3 (2.2)
Computed tomography scan	4 (2.8)	1 (0.7)
Not confirmed/not evaluable	6 (4.2)	9 (6.7)
*Anatomical extent of proximal DVT, n (%)*		
Limited (popliteal vein or more distal)	25 (17.5)	17 (12.7)
Intermediate (most proximal: superficial femoral vein)	25 (17.5)	27 (20.1)
Extensive (most proximal: common femoral or iliac vein)	112 (78.3)	108 (80.6)
*Time from onset of symptoms to randomization, days*		
Median	9.0	9.0
Interquartile range	4.0–19.0	4.0–20.0
*Cause of DVT or PE, n (%)*^*†*^		
Unprovoked	164 (74.5)	162 (74.0)
Secondary DVT or PE	56 (25.5)	57 (26.0)
Recent surgery or trauma	34 (15.5)	39 (17.8)
Immobilization	21 (9.5)	24 (11.0)
Estrogen therapy	2 (0.9)	4 (1.8)
Active cancer	6 (2.7)	7 (3.2)
Puerperium	2 (0.9)	4 (1.8)
Previous DVT or PE	27 (12.3)	36 (16.4)

*Plus–minus values are means ± SD. There were no significant differences between the two study groups. Percentages may not total 100 because of rounding.

^†^Patients could have multiple causes of DVT or PE.

DVT, deep-vein thrombosis; PE, pulmonary embolism; SD, standard deviation.

### Treatment and follow-up

In the standard-therapy group, the median duration of enoxaparin treatment was 9 days (interquartile range, 7–12), and the INR at the end of enoxaparin treatment was ≥2.0 in 84.9% of patients. The mean percentage of time during which the INR was in the therapeutic range (2.0–3.0) was 52.4%; the corresponding mean percentages for an INR >3.0 and <2.0 were 10.1% and 37.5%, respectively. The mean percentage of time within the therapeutic range ranged from 45.6% (during the third month) to 68.3% (during month 7). In the rivaroxaban group, adherence to therapy was >80% in 93.2% of patients. As an event-driven study, termination upon the required number of events resulted in treatment duration being less than intended in 14 (6.4%) patients in the rivaroxaban group and 11 (5.0%) patients in the standard-therapy group. Four (1.8%) patients in the rivaroxaban group and three (1.4%) patients in the standard-therapy group were lost to follow-up (Table 2).

**Table 2:** Characteristics associated with anticoagulant treatment

**Characteristic**	**Rivaroxaban (n=220)**	**Standard therapy (n=219)**
*At least one dose of a study drug received, n (%)*	219 (99.5)	218 (99.5)
*Intended duration of treatment, n (%)*		
3 months	56 (25.5)	55 (25.1)
6 months	131 (59.5)	131 (59.8)
12 months	33 (15.0)	33 (15.1)
*Mean study duration, days*	198.5	191.3
Mean study treatment duration, days	162.2	155.2
*Reasons for premature discontinuation of treatment, n (%)*		
Any reason	27 (12.3)	37 (16.9)
Adverse event	14 (6.4)	15 (6.8)
Consent withdrawn	5 (2.3)	14 (6.4)
Loss to follow-up	4 (1.8)	3 (1.4)

### Clinical outcomes

The clinical outcomes are shown in Table 3. The primary efficacy outcome occurred in seven (3.2%) of the 220 patients in the rivaroxaban group compared with seven (3.2%) of the 219 patients in the standard-therapy group, for a hazard ratio of 1.04 (95% confidence interval [CI] 0.36–3.0). By day 21, at the end of twice-daily rivaroxaban administration, the primary efficacy outcome had occurred in one (0.5%) patient in the rivaroxaban group and in four (1.8%) patients in the standard-therapy group.

**Table 3:** Clinical outcomes

**Outcome**	**Rivaroxaban**	**Standard therapy**
**Efficacy**		
*Intention-to-treat population, n*	220	219
*Recurrent VTE, n (%)*	7 (3.2)	7 (3.2)
*Type of first recurrent VTE, n*		
Fatal PE	0	0
Death in which PE could not be ruled out	1	0
Non-fatal PE	2	2
Recurrent DVT	4	5
*Net clinical benefit: VTE plus major bleeding, n (%)*	7 (3.2)	12 (5.5)
**Safety**		
*No. of patients, safety population*	219	218
*First episode of major or clinically relevant non-major bleeding during treatment, n (%)*	13 (5.9)	20 (9.2)
*Major bleeding episode, n (%)*	0	5 (2.3)
Fatal	0	2
Gastrointestinal	0	1
Intracranial	0	1
Other non-fatal episode in a critical site	0	1
Intracranial	0	1
Associated with a fall in hemoglobin of ≥2 g/dL, transfusion of ≥2 units, or both	0	2

DVT, deep-vein thrombosis; PE, pulmonary embolism; VTE, venous thromboembolism.

**Figure 1:**
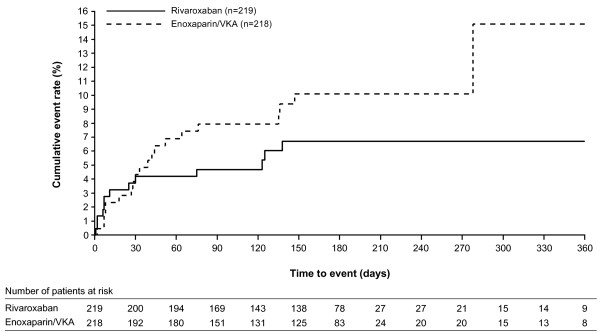
**Kaplan–Meier curve for cumulative event rate for treatment-emergent major and clinically relevant non-major bleeding.** VKA, vitamin K antagonist.

The principal safety outcome, a first major or clinically relevant non-major bleeding episode, occurred in 13 (5.9%) of the 219 patients in the rivaroxaban group compared with 20 (9.2%) of the 218 patients in the standard-therapy group (hazard ratio, 0.63; 95% CI 0.31–1.26; p=0.19) (Figure 1). Major bleeding occurred in none of the patients in the rivaroxaban group and in five (2.3%) patients in the standard-therapy group. Of these five major bleeding events, two were fatal.

Among patients who were older than 75 years, major or clinically relevant non-major bleeding occurred in three (9.1%) of the 33 rivaroxaban recipients compared with six (21.4%) of the 28 standard-therapy recipients. In those patients with CrCl <50 mL/min, this outcome occurred in one (5.3%) and six (21.4%) of the 19 and 28 patients, respectively. In frail patients (defined as age >75 years, CrCl <50 mL/min, and/or body weight ≤50 kg), major or clinically relevant non-major bleeding occurred in four (8.9%) of the 45 rivaroxaban patients compared with seven (15.2%) of the 46 standard-therapy patients (Table 4).

**Table 4:** Incidence of major and clinically relevant non-major bleeding according to subgroups

		**Rivaroxaban n/N (%)**	**Standard therapy n/N (%)**
*Age, years*	<65	7/134 (5.2)	5/133 (3.8)
	65–75	3/52 (5.8)	9/57 (15.8)
	>75	3/33 (9.1)	6/28 (21.4)
*Creatinine clearance, mL/min*	≥80	8/117 (6.8)	4/116 (3.4)
	50–<80	3/75 (4.0)	10/72 (13.9)
	<50	1/19 (5.3)	6/28 (21.4)
*Body weight, kg*	≤50	0/3	2/11 (18.2)
	>50–70	9/134 (6.7)	14/134 (10.4)
	>70–90	3/79 (3.8)	4/69 (5.8)
	>90	0/1	0/4
Fragility*	No	9/174 (5.2)	13/172 (7.6)
	Yes	4/45 (8.9)	7/46 (15.2)

*Defined as age >75 years, creatinine clearance <50 mL/min, and/or body weight ≤50 kg.

### Other outcomes

Net clinical benefit (i.e. the combination of the primary efficacy outcome and major bleeding) was more favorable in the rivaroxaban group: the net clinical benefit outcome occurred in seven (3.2%) of the 220 rivaroxaban recipients versus 12 (5.5%) of the 219 standard-therapy recipients, for a hazard ratio of 0.59 (95% CI 0.23–1.51) (Table 3). Two (0.9%) patients in the rivaroxaban group developed another vascular outcome (both ischemic strokes), compared with two (0.9%) patients in the standard-therapy group (ischemic stroke and myocardial infarction). Six (2.7%) patients in the rivaroxaban group and 11 (5.0%) patients in the standard-therapy group died during the study.

## Discussion

This analysis of data from Chinese patients included in the EINSTEIN DVT and EINSTEIN PE studies [11] showed similar point estimates of relative efficacy and safety of oral rivaroxaban alone compared with standard-therapy for the treatment of symptomatic DVT and/or PE, consistent with results observed in the global studies.

There is an indication that VKAs are dosed more cautiously in China; the percentage of time spent with INR value <2.0 was higher compared with global studies (37% vs. 22%) and the percentage of time spent in the therapeutic range was lower (52% vs. 62%). Interestingly, the incidence of major or clinically relevant non-major bleeding was similar in China compared with the global study population (9.2% vs. 10.0%) for standard-therapy recipients, whereas this incidence tended to be lower (5.9% vs. 9.4%) for rivaroxaban recipients [11]. This confirms the favorable safety profile of oral rivaroxaban (compared with standard therapy for the treatment of VTE) in Chinese patients.

With regard to efficacy, recurrent events occurred in 3.2% of patients in the rivaroxaban group and 3.2% of those in the standard-therapy group. These rates were slightly higher than in the global study (2.1% and 2.3% for rivaroxaban and standard therapy, respectively).

There has been a traditional perception that, because of ethnic differences, Chinese people do not develop VTE at the same rate as Caucasians. However, although incidences of DVT and PE in the general Chinese population are unknown, some published studies suggest that the incidence of VTE in hospitalized medically ill patients is similar in these ethnic groups [12,13]. Regardless of the rates of VTE, the standard of care in China is the same as the standard therapy used in the EINSTEIN program, i.e. low-molecular-weight heparin overlapping with and followed by an adjusted-dose VKA. Consistent with the global data, rivaroxaban showed similar efficacy to standard therapy in the Chinese patient population, with a tendency towards an improved safety profile, as demonstrated by the lower incidence of major and clinically relevant non-major bleeding complications, a result that was also observed in frail patients.

A number of studies have shown that Chinese patients have lower warfarin requirements to achieve a target INR range, indicating that Chinese patients may have an increased sensitivity to the anticoagulant effect of VKAs compared with Caucasians [14]. This sensitivity to warfarin has not been definitively linked to higher rates of bleeding in Chinese patients, and little is known about the optimal intensity of anticoagulation. The few studies that have assessed whether bleeding on anticoagulation is similar in Chinese and Caucasian patients have shown mixed results, were retrospective, and included small numbers of patients [15,16]. In the global studies, there is an indication that Chinese doctors give VKAs in dosages aimed to obtain a slightly lower INR. This did not affect the more favorable bleeding profile of rivaroxaban.

## Conclusions

In summary, the results of this analysis of the patients included in China in the EINSTEIN DVT and EINSTEIN PE trials show that the performance of rivaroxaban was consistent with that observed in the global population, with a similar efficacy and a better bleeding profile to standard therapy of enoxaparin overlapping with and followed by a VKA. The data indicate that oral rivaroxaban provides a new, improved option for the treatment of DVT and PE in Chinese patients.

## Abbreviations

CI: Confidence interval; CrCl: Creatinine clearance; CYP: Cytochrome P450; DVT: Deep-vein thrombosis; INR: International normalized ratio; PE: Pulmonary embolism; VKA: Vitamin K antagonist; VTE: Venous thromboembolism.

## Competing interests

MHP: has acted as a consultant for Bayer HealthCare, Sanofi-Aventis, Boehringer Ingelheim, GlaxoSmithKline, Daiichi Sankyo, Leo Pharma, ThromboGenics, and Pfizer. ILM, AFP, KM, and AWAL are employees of Bayer Pharma AG. The other authors have no competing interests to declare.

## Authors’ contributions

YW, CW, ILM, MHP, and AWAL interpreted the data and were involved in the initial drafting and revising of the manuscript; AFP and KM performed the statistical analyses and contributed to the discussions of the data. All authors contributed to the execution of the study and discussed the study results. All authors provided final approval for publication of the manuscript.

## Supplementary Material

Additional file 1 **The Chinese EINSTEIN Investigators.** *Investigator included in the author list was involved in developing the manuscript draft. Click here for file
