# Supplementary material for: Rivaroxaban for the treatment of symptomatic deep-vein thrombosis and pulmonary embolism in Chinese patients: a subgroup analysis of the EINSTEIN DVT and PE studies
Source: Thromb J. 2013 Dec 16;11:25. doi: 10.1186/1477-9560-11-25 (PMC3896794; doi:10.1186/1477-9560-11-25)
Supplement: Additional file 1 — The Chinese EINSTEIN Investigators. *Investigator included in the author list was involved in developing the manuscript draft. [file 1477-9560-11-25-S1.docx]

| **No.** | **Investigator sites** | **Investigators** | **Number of patients enrolled** |
| --- | --- | --- | --- |
| **1** | Department of Vascular Surgery, Zhongshan Hospital Fudan University | Yuqi Wang* | 28 |
| **2** | Beijing Institute of Respiratory Medicine, Beijing ChaoYang Hospital | Chen Wang* | 40 |
| **3** | Department of Vascular Surgery, Beijing Anzhen Hospital | Zhong Chen* | 49 |
| **4** | Department of General Surgery, Peking Union Medical College Hospital | Changwei Liu* | 23 |
| **5** | Department of Cardiology, Peking University People's Hospital | Juntang Xu | 9 |
| **6** | Department of Cardiology, Fuwai Hospital | Zhihong Liu* | 34 |
| **7** | Department of Vascular Surgery, Wuhan Union Hospital, Tongji Medical College, Huazhong University of Science and Technology | Bi Jin* | 40 |
| **8** | Department of Pulmonary Circulation, Shanghai Pulmonary Hospital | Zhicheng Jing* | 19 |
| **9** | Department of Respiratory Medicine, Beijing Shijitan Hospital | Yingmin Ma | 12 |
| **10** | Department of Respiratory Medicine, The 2nd Affiliated Hospital of Harbin Medical University | Yuxia Shao* | 27 |
| **11** | Department of Respiratory Medicine, Sir Run Run Shaw Hospital, Zhejiang University School of Medicine | Kejing Ying* | 31 |
| **12** | Department of Respiratory Medicine, The First Affiliated Hospital of Guangzhou Medical University | Rongchang Chen | 12 |
| **13** | Department of Respiratory, Beijing Anzhen Hospital | Shuang Liu | 20 |
| **14** | Department of Respiratory Medicine, The First Affiliated Hospital of Sun Yat-Sun University | Canmao Xie | 5 |
| **15** | Department of Respiratory Medicine, The People's Hospital of Guangxi Zhuang Autonomous Region | Zhiqiang Qin | 19 |
| **16** | Department of Vascular Surgery, The Affiliated Renji Hospitial of Shanghai Jiao Tong University School of Medicine | Jiwei Zhang* | 37 |
| **17** | Department of Cardiology, The First Affiliated Hospital of Sun Yat-Sun University | Shenming Wang, Yugang Dong | 12 |
| **18** | Department of Vascular Surgery, PLA General Hospital | Wei Guo | 9 |
| **19** | Department of Vascular Surgery, The People's Hospital of Liaoning Province | Danming Wu | 7 |
| **20** | Department of Vascular Surgery, Shanghai Changhai Hospital | Zaiping Jing | 3 |
| **21** | Department of Vascular Surgery, The Second Affiliated Hospital of Suzhou University | Xiaoqiang Li | 4 |

**Additional file 1** *Investigator on author list as also involved in developing draft of manuscript
